# IgPose: a generative data-augmented pipeline for robust immunoglobulin–antigen binding prediction

**DOI:** 10.1093/bioinformatics/btag076

**Published:** 2026-02-15

**Authors:** Tien-Cuong Bui, Injae Chung, Wonjun Lee, Junsu Ko, Juyong Lee

**Affiliations:** R&D Department, Arontier Co., Ltd., Seoul, 06735, Republic of Korea; R&D Department, Arontier Co., Ltd., Seoul, 06735, Republic of Korea; R&D Department, Arontier Co., Ltd., Seoul, 06735, Republic of Korea; R&D Department, Arontier Co., Ltd., Seoul, 06735, Republic of Korea; R&D Department, Arontier Co., Ltd., Seoul, 06735, Republic of Korea; Department of Molecular Medicine and Biopharmaceutical Sciences, Graduate School of Convergence Science and Technology, Seoul National University, Seoul, 08826, Republic of Korea; Research Institute of Pharmaceutical Science, College of Pharmacy, Seoul National University, Seoul, 08826, Republic of Korea

## Abstract

**Motivation:**

Predicting immunoglobulin–antigen (Ig–Ag) binding remains a significant challenge due to the paucity of experimentally resolved complexes and the limited accuracy of *de novo* Ig structure prediction.

**Results:**

We introduce IgPose, a generalizable framework for Ig–Ag pose identification and scoring, built on a generative data-augmentation pipeline. To mitigate data scarcity, we constructed the Structural Immunoglobulin Decoy Database (SIDD), a comprehensive repository of high-fidelity synthetic decoys. IgPose integrates equivariant graph neural networks, ESM-2 embeddings, and gated recurrent units to synergistically capture both geometric and evolutionary features. We implemented interface-focused *k*-hop sampling with biologically guided pooling to enhance generalization across diverse interfaces. The framework comprises two sub-networks—IgPoseClassifier for binding pose discrimination and IgPoseScore for DockQ score estimation—and achieves robust performance on curated internal test sets and the CASP-16 benchmark compared to physics and deep learning baselines. IgPose serves as a versatile computational tool for high-throughput antibody discovery pipelines by providing accurate pose filtering and ranking.

**Availability and implementation:**

IgPose is available on GitHub (https://github.com/arontier/igpose).

## 1 Introduction

Immunoglobulin–antigen (Ig–Ag) recognition is a cornerstone of adaptive immunity (Lu *et al.* 2018) and underpins the development of many therapeutic biologics (Chan *et al.* 2025), molecular diagnostics (García-Fiñana and Buchan 2021), and vaccine development (Crank *et al.* 2019). Accurate structure modeling of Ig–Ag complexes, identifying correct spatial orientations and binding poses, and estimating binding affinities are critical for the rational design of therapeutic antibodies (Norman *et al.* 2020). However, this task remains challenging because of the limited number of experimentally resolved Ig–Ag structures, the conformational plasticity of complementarity-determining region (CDR) loops, and the high-dimensional, heterogeneous nature of protein–protein interfaces (PPI). Collectively, these factors hinder the out-of-distribution generalization of existing computational models, often resulting in the failure of conventional interface scoring functions applied to novel epitope landscapes.

Traditional physics-based tools such as Rosetta (Alford *et al.* 2017) and Prodigy (Xue *et al.* 2016) offer interpretable energy-based scoring terms for PPI but frequently underperform when distinguishing near-native from non-native Ig–Ag binding poses at scale. Recent deep learning (DL) methods for protein–protein pose classification and scoring, such as TRScore (Guo *et al.* 2022), GNN-DOVE (Wang *et al.* 2021), DeepRank-GNN-ESM (Xu and Bonvin 2024), and ProAffinity-GNN (Zhou *et al.* 2024), improve scoring by learning from interface representations, but typically lack geometric equivariance and often exhibit overfitting and/or reduced performance on unseen structures. Recently, an antibody-specific B cell epitope prediction tool, AbEpiTope-1.0 (Clifford *et al.* 2025), was proposed to use AlphaFold-2 Multimer (AFM) (Evans *et al.* 2021) for structural modeling and ESM-IF embeddings (Hsu *et al.* 2022) pooled over Ig–Ag interface nodes with a shallow multi-layer perceptron (MLP) to discriminate native-like Ig–Ag poses and estimate interface quality. However, varying performance across different benchmarks suggests their limited generalizability.

**Figure 1:**
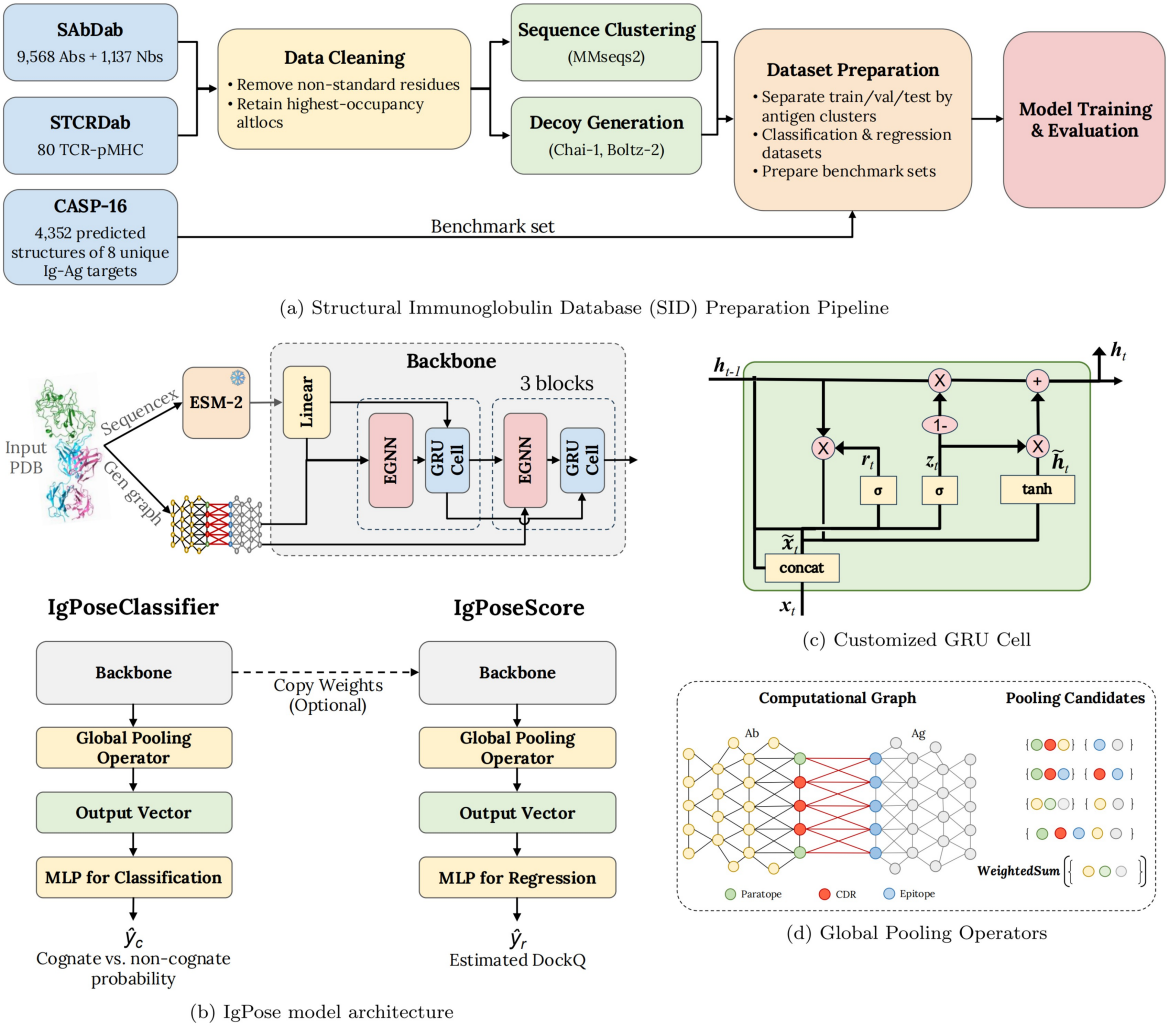
An overview of IgPose model architecture and its internal components. (a) The data preparation pipeline from data collection, data cleaning, and decoy generation with Chai-1 (Discovery *et al*. 2024) and Boltz-2 (Passaro *et al*. 2025, Wohlwend *et al*. 2025) to data preparation and model training and evaluation. (b) The IgPose—equivariant message-passing architecture built upon EGNN (Satorras et al. 2021) and a customized GRU module. (c) The architecture of the customized GRU module. (d) Alternative global pooling operators corresponding to different selective regions.

Here, we introduce IgPose, an Ig–Ag complex scoring model based on a generative framework that integrates evolutionary context, geometric inductive bias, structural augmentation, and task-specific learning objectives (Fig. 1). IgPose enriches contextual features with evolutionary knowledge from the ESM-2 embeddings (Lin *et al.* 2023) of Ig and Ag sequences and uses E(n)-equivariant graph neural networks (EGNN) (Satorras *et al.* 2021) coupled with a customized gated recurrent unit (GRU) module (Cho *et al.* 2014) to model long-range interactions while preserving physical symmetries. To overcome the scarcity of experimentally determined Ig–Ag structures in public repositories, we introduced a generative pipeline to supplement the training set with various synthetic decoy structures modeled using Chai-1 (Discovery *et al.* 2024) and Boltz-2 (Passaro *et al.* 2025, Wohlwend *et al.* 2025). Our framework uses two core models, IgPoseClassifier and IgPoseScore, to address both Ig–Ag pose classification and regression tasks. IgPoseClassifier performs binary classification of a given Ig–Ag structure into native-like or non-native conformations. IgPoseScore estimates the DockQ score (Basu and Wallner 2016) of a complex model structure. We design task-specific loss functions for each tool to facilitate efficient model training.

Our evaluation results demonstrate that IgPose consistently outperforms traditional physics-based methods (Xue *et al.* 2016, Alford *et al.* 2017) and existing DL models (Wang *et al.* 2021, Guo *et al.* 2022, Zhou *et al.* 2024) across various benchmark sets. Inconsistent performance trends of baseline methods emphasize their limitations in generalizability under distribution shifts and structural variations. In contrast, IgPose achieves up to a two-fold improvement in Area Under the Precision-Recall Curve (AP) scores across diverse benchmarks, which we attribute to the integration of our generative data-augmentation strategy with a hybrid architecture combining EGNNs, evolutionary ESM-2 embeddings, and GRUs to capture complex interfacial features. These results position IgPose as a robust computational framework for antibody discovery, facilitating more accurate discrimination and ranking of lead candidates during virtual screening.

## 2 Experimental methods

### 2.1 Problem formulation

Let *S* denote an Ig–Ag complex provided in PDB format, containing antibody chain(s) and one antigen chain. We represent *S* as a computational graph $G=\left(V,E,X_{v},X_{e},P\right)$, where $V={\left\{v_{i}\right\}}_{i=1}^{N}$ is a set of *N* amino‐acid residues, E is a set of connections between them, and $P=\{p_{a}\in R^{3}|a\in \overset{N}{\underset{i=1}{\cup }}\text{atoms}(v_{i})\}$ collects the 3D positions of all atoms. We seek two functions.

First, a classifier $f_{\theta }$ is defined as follows:


$$f_{\theta }:G\to [0,1],\;{\hat{y}}_{c}=f_{\theta }(G),$$

where ${\hat{y}}_{c}$ is the probability that *S* is a native-like binding pose of a cognate Ig–Ag pair (y=1) versus a non-native decoy (y=0) including a non-native bound pose of the cognate Ig–Ag pair or any bound pose of a non-cognate Ig–Ag pair. Second, a regressor $r_{\phi }$ is defined as follows:


$$r_{\phi }:G\to R,\;{\hat{y}}_{r}=r_{\phi }(G),$$

where ${\hat{y}}_{r}$ is an estimated DockQ score of a given model complex structure.

### 2.2 Data sources and curation

We assembled the *Structural Immunoglobulin Database* (SID), a comprehensive structural dataset of Ig–Ag complexes by combining experimentally determined structures from public repositories with systematically generated decoys.

*Structural Antibody Database (SAbDab):* X-ray crystallographic (XRC) and electron cryomicroscopy (cryo-EM) structures of antibodies (Ab; 4362 structures) and nanobodies (Nb; 1137 structures) bound to *monomeric* (single chain) antigens, deposited prior to 23 September 2024, were curated from SAbDab (Dunbar *et al.* 2014). During data cleaning, non-standard amino acids with *HETATM* records were removed. When residues had alternative locations (*altlocs*), the one with the highest occupancy was retained. To generate training clusters, antigen sequences—derived from *SEQRES* records or the UniProt database (Bateman *et al*. 2025)—were clustered by 30% sequence similarity and 30% sequence coverage using MMseqs2 (Steinegger and Söding 2017, 2018), yielding 942 clusters comprising 5499 Ig–Ag pairs.

*Structural T-Cell Receptor Database (STCRDab):* A curated set of high-resolution, annotated structures of T-cell receptors (TCR) bound to peptide-MHC (major histocompatibility complex; pMHC) complexes from STCRDab (Leem *et al.* 2018) were fetched from Zhang *et al.* (2023). Structures were similarly cleaned to remove non-standard amino acids and low occupancy *altlocs*. A total of 80 TCR-pMHC complexes were clustered by MHC class (I or II).

*Structural Immunoglobulin Decoy Dataset (SIDD):* Based on the structural data curated from SAbDab and STCRDab, we constructed SIDD, an *in-house* database of decoy Ig–Ag structures generated using biomolecular structure prediction tools, Chai-1 (Discovery *et al.* 2024) and Boltz-2 (Passaro *et al.* 2025, Wohlwend *et al.* 2025).

1. *Cognate Ig–Ag decoys:* an *in silico* generated dataset composed of re-predicted structures of cognate Ab-Ag, Nb-Ag, and TCR-pMHC pairs, yielding *ca.* $9.2\times 10^{3}$ structures. A DockQ score of 0.8 (*high-quality*) (Basu and Wallner 2016) was used as a threshold to discriminate between *positive* (≥0.8) and *negative* (<0.8) decoy structures.
2. *Non-cognate Ig–Ag decoys:* an *in silico* generated dataset composed of Abs, Nbs, or TCRs in complex with non-cognate antigens. Ig and Ag sequences were clustered by 100 and 90% similarity, respectively, and 80% coverage using MMseqs2 (Steinegger and Söding 2017, 2018). One sample was selected per cluster to remove redundancy. To avoid potential artifacts arising from matching antigens (i.e. false positives), we purposefully paired Abs and Nbs with pMHCs, and TCRs with monomeric antigens, generating *ca.* $10^{5}$  *negative* decoy structures.

The generated structures were clustered following the same sequence-based clustering procedure used for the SAbDab and STCRDab datasets, based on their corresponding target antigen (monomeric antigen or pMHC).

### 2.3 Benchmark dataset

*Critical Assessment of Structure Prediction 16 (CASP-16):* We collated all structure predictions submitted for eight Ig–Ag docking targets released at the CASP-16 competition (CASP-16 2024) (predictioncenter.org/casp16): *H1204* (PDB-8vyl), *H1215* (unreleased), *H1222* (PDB-9cqd), *H1223* (PDB-9cqb), *H1225* (PDB-9cqa), *H1232* (PDB-9cn2), *H1233* (PDB-9cbn), and *H1244* (unreleased). These complexes, which include Abs, Nbs, and single-chain variable fragments (scFvs) bound to their cognate antigens, are experimentally resolved but unpublished (at the time of the competition), ensuring that their 3D structures are novel and “unseen” by existing computational prediction methods, including IgPose. To ensure there was no data leakage between our training cut-off (2024.09.23) and the CASP-16 modeling end date (2024.08.31), we cross-checked all eight CASP-16 targets against our curated dataset and confirmed that none were present. During data cleaning, each Ig copy in a structure was paired with its bound antigen(s). Similar to SIDD, we used a DockQ score (Basu and Wallner 2016) of 0.8 to discriminate between *positive* and *negative* decoy structures; DockQ scores were provided by the CASP-16 assessors (CASP-16 2024). The CASP-16 dataset is composed of 4352 predicted structures.

### 2.4 Dataset grouping

We grouped *SID* into three internal datasets for classification and regression tasks. Table 1 presents statistics for these datasets and the CASP-16 benchmark. The first classification dataset (SID-CA) comprises all experimentally determined “native” Ig–Ag structures drawn from SAbDab and STCRDab, along with cognate and non-cognate conformations generated by Chai-1 (Discovery *et al.* 2024). To mitigate bias from antigen homology, samples were stratified by antigen clusters into training, validation, and test splits in an approximate 6:2:2 ratio. To investigate generalization across alternative structure prediction tools, we designated a second classification test set (SID-CB) for structures generated exclusively with Boltz-2 (Passaro *et al.* 2025, Wohlwend *et al.* 2025).

**Table 1:** Statistics of our Structural Immunoglobulin Database (SID) and CASP-16 benchmark dataset.^a^

Dataset	Split	#Positive	#Negative
SID-CA	Train	3668	68907
	Validation	1445	9133
	Test	928	17092
SID-CB	Test	1001	16436
SID-R	Train	4668	10599
	Validation	1921	1448
	Test	1025	1116
CASP-16	Benchmark	349	4003

a Here, *positive* represents conformations that are either experimentally resolved “native” complexes or cognate decoys with DockQ scores ≥0.8; *negative* indicates structural decoys with DockQ scores <0.8. SID-C and SID-R denote datasets for classification and regression tasks, respectively. SID-CA and SID-CB are two classification datasets containing decoy conformations generated by Chai-1 and Boltz-2, respectively.

To train a regression model estimating DockQ scores of cognate decoys, we constructed a regression dataset (SID-R) which integrates experimentally resolved structures from SAbDab and STCRDab, and Chai-1- and Boltz-2-generated decoy structures in SIDD. Each structure is annotated with a continuous DockQ label (SAbDab, STCRDab = 1; non-cognate decoys = 0; cognate decoys ∈(0,1)). To maintain an unbiased mix of *negative* samples, we randomly sampled seven Chai-1 decoys per Ig chain within each antigen cluster. SID-R was then split by the same cluster IDs used in SID-C into training, validation, and test subsets.

Finally, we used the CASP-16 Ig–Ag prediction results for external validation of both tasks. In the classification setting, we adopted the same DockQ threshold of 0.8 for *positive*/*negative* labeling to ensure that only the highest-quality docking poses are labeled as *positive*. This stringent cut-off guards against cases where strong signals on non-Ig–Ag interfaces can inflate the overall (global) DockQ score, thus excluding conformations with suboptimal Ig–Ag contacts. In the regression setting, we instead predicted continuous DockQ values directly against the scores obtained by the assessors of CASP-16.

### 2.5 CDR annotation

ANARCI (Dunbar and Deane 2016) was used to annotate and extract information about complementarity determining regions (CDRs) from Ab, Nb, scFv, and TCR sequences. The Chothia numbering scheme (Chothia and Lesk 1987, Al-Lazikani *et al.* 1997) was used to annotate Abs, Nbs, and scFvs, and the IMGT numbering scheme (Lefranc *et al.* 1999, 2003, Lefranc 2015) for TCRs.

### 2.6 Graph construction and features

Ig–Ag structures in PDB or mmCIF formats were converted into PDBQT files using MGLtools (Morris *et al.* 2009). During this process, implicit (non-polar) hydrogens were removed, and explicit (polar) hydrogens were introduced where absent. For graph construction, we defined edge sets as follows: $E_{r}=\{(i,j)|\min_{a\in A_{i},\;b\in A_{j}}∥p_{a}-p_{b}{∥}_{2}\leq \tau_{r}\},$ where A is the set of atoms of the corresponding residue, $p\in R^{3}$ denotes the Cartesian coordinate, and r∈{intra,inter}. Specifically, $E_{\text{intra}}$ is the intra-residue edge set including connections between nodes within antibody/antigen residues s.t. $\tau_{\text{intra}}\leq 3.5Å$, while $E_{\text{inter}}$ includes inter-molecular edges at the binding interface s.t $\tau_{\text{inter}}\leq 10.0Å$.

Input node features $X_{v}\in R^{N\times d_{x}}$ were obtained from ESM-2 (Lin *et al.* 2023), where $d_{x}=320$. Input edge attributes $X_{e}\in R^{N\times d_{e}}$ comprise three raw distances (minimum atomic distance, Cα-Cα distance, and center-of-mass distance), expanded via a Gaussian RBF map $\phi_{D}:R\to R^{D}$ with *D* log-spaced scales in [0.25,8], yielding a $d_{e}=3\times D$. We used D=10 by default and varied *D* in ablations.

### 2.7 Model architecture

Here, we present our equivariant graph neural network (EGNN) architecture (Fig. 1). Let the input graph be $G=(V,E,X_{v},X_{e},P)$. The network comprises *T* EGNN (Satorras *et al.* 2021) layers, each interleaved with a custom GRU gate (Fig. 1c), followed by a weighted pooling head and an MLP classifier. We map node features $X\in R^{N\times d_{x}}$ into a hidden space:


(1) $$H^{\left(0\right)}=\text{SiLU}\left(XW_{\text{in}}+b_{\text{in}}\right)\in R^{N\times d_{h}},$$

For t=1,…,T, we apply:


(2) $$\begin{array}{c} {\tilde{H}}^{\left(t\right)},P^{\left(t\right)}=\text{EGNN}(H^{\left(t-1\right)},P^{\left(t-1\right)}),\\ H^{\left(t\right)}=\text{GRU}([{\tilde{H}}^{\left(t\right)}\;|\;H^{\left(t-1\right)}],H^{\left(t-1\right)}). \end{array}$$

Integrating the GRU function (Xiong *et al.* 2020, Zhou *et al.* 2024) into GNNs is prevalent as it acts as a learnable gated residual update. As *H* is an invariant feature in EGNN (Satorras *et al.* 2021), adding a gated connection on top does not break the equivariant properties of P. In our design, the GRU function introduces an additional residual connection to ease the oversmoothing problem of GNNs by concatenating $H^{\left(t-1\right)}$ and ${\tilde{H}}^{\left(t\right)}$ as the input state. This modification enforces a stronger self-loop to preserve a node’s identity in message passing steps. It also introduces a direct linear path for gradients to flow backward to $H^{\left(t-1\right)}$ via the candidate gate *n* even when other gates saturate. Further detail can be found in the Supplementary Information.

After *T* equivariant message-passing iterations on G, we perform a read-out function on a selected subset S∈V. The perturbation approach, exploring a set of essential node embeddings maximizing GNN performance, is common practice in explainable artificial intelligence (XAI) research (Bui and Li 2023, Bui *et al.* 2023). In practice, we can perform this procedure either through sampling-based and learning-based algorithms or with domain expertise. Given the enormous size of computational graphs and specificity of the Ig–Ag binding problem, we opt for the latter approach and introduce several strategies to define S: *All nodes—*pooling all nodes, *Interface Only—*pooling only binding-interface nodes, *CDR-Epitope Only—*pooling only nodes in edges between CDRs and epitopes, *CDR Only—*pooling only nodes in CDRs, *w/o interface—*pooling all nodes except those in inter Ig–Ag edges, *w/o CDR-Epitope—*pooling all nodes except those in edges between CDRs and epitopes, *w/o CDR—*pooling all nodes excluding those in CDRs (Fig. 1d). Node weights and the global graph embedding are then:


(3) $$w_{i}=\text{sigmoid}\left(w_{p}^{⊤}h_{i}+b_{p}\right),\;g=\sum_{i\in S}w_{i}h_{i},$$

with |S|≤N, where $h_{i}$ corresponds to a row *i* in $H^{\left(T\right)}$.

Our architecture supports both classification and regression tasks. We train classification models by minimizing the classification objective function on discrete labels. After that, we initialize a regression model from a trained classifier by replacing the softmax output with a linear layer and fine-tuning all parameters on continuous labels using a regression loss function. This transfer learning strategy improves regression performance with fewer data samples. For the readout layers, the following layers are used.

IgPoseClassifier—the classifier—uses a two-layer MLP with SiLU activation and softmax:


(4) $${\hat{y}}_{c}=\text{Softmax}\left(W_{c}^{\left(2\right)}\text{SiLU}(W_{c}^{\left(1\right)}g+b_{c}^{\left(1\right)})+b_{c}^{\left(2\right)}\right).$$

IgPoseScore—the regressor—estimates DockQ scores and substitutes the classifier head with a scaled-tanh predictor:


(5) $${\hat{y}}_{r}=\frac{1}{2}\left(\tanh(0.5\times z)+1\right),\;z=W_{r}g+b_{r}.$$

As deep ensembles (Lakshminarayanan *et al.* 2017) can improve predictive uncertainty estimation and robustness, we define a weighted ensemble function to aggregate the predicted results of *M* models into ${\hat{y}}^{*}$, as follows:


(6) $${\hat{y}}^{*}=\sum_{m=1}^{M}w_{m}\hat{y},\;\text{where}\;\sum_{m=1}^{M}w_{m}=1,\;w_{m}\geq 0.$$

### 2.8 Training objective functions

We optimize specific objectives for each task. For classification, we learn θ by minimizing:


(7) $$\theta^{*}=\arg\underset{\theta }{\min}L_{c}(\theta ),$$

(8) $$L_{c}=L_{\text{GC}}+\alpha L_{\text{NC}}+\beta L_{\text{MDN}}.$$

Here, $L_{\text{GC}}$ is the graph-level cross-entropy loss that predicts the class of an input graph G, $L_{\text{NC}}$ the node-level cross-entropy that predicts individual node types (e.g. antigen, heavy chain, light chain), and $L_{\text{MDN}}$ the negative Pearson correlation between predicted probability and ground-truth DockQ. The Lagrange multipliers set as $\alpha =10^{-3}$ and $\beta =2\times 10^{-3}$. For regression, we optimize:


(9) $$\phi^{*}=\arg\underset{\phi }{\min}L_{r}(\phi ),$$

(10) $$L_{r}=L_{\text{coeff}}+L_{\text{rank}}.$$

Here, $L_{\text{coeff}}=-\text{Corr}(y,{\hat{y}}_{r})$ for maximizing Pearson correlation (Shen *et al.* 2023), and $L_{\text{rank}}$ is the listwise ranking loss (Cao *et al.* 2007).

### 2.9 Training and evaluation protocol

*Baselines:* We benchmarked our models against MIEnsembles [the highest computed AUC, AP and *r* scores on CASP-16 (CASP-16 2024)], standard physics-based scoring functions [Prodigy (Xue *et al.* 2016) and Rosetta (Alford *et al.* 2017)] and publicly available DL models [TRScore (Guo *et al.* 2022), GNN‐DOVE (Wang *et al.* 2021), DeepRank‐GNN‐ESM (DR-GNN-ESM) (Xu and Bonvin 2024), ProAffinity‐GNN (Zhou *et al.* 2024), and AbEpiTope-1.0 (Clifford *et al.* 2025)]. All DL models are executed using their default configurations.

*Evaluation Metrics:* We independently evaluated the classification and regression performance. For classification, we report five standard metrics: *Precision* (P), *Recall* (R), *F1-score* (F1), *the Area Under the Receiver Operating Characteristic Curve* (AUC), and *the Area Under the Precision-Recall Curve* (AP).

For regression, we quantify the agreement between the predicted values ${\hat{y}}_{i}$ and the true targets $y_{i}$ using the Pearson correlation coefficient (*r*).

*IgPose Implementations:* Processing an entire graph G is suboptimal in both prediction performance and computational efficiency. Therefore, we extracted a subgraph $G_{s}$ via 3-hop sampling (Algorithm 1, available as supplementary data at *Bioinformatics* online) around interface edges $E_{\text{inter}}$, restricting message-passing to relevant binding-site neighborhoods. Specifically, we implemented two alternative sampling strategies: starting from unique nodes in $E_{\text{inter}}$ or from nodes in CDRs, with both procedures stopping once $G_{s}$ reached a pre-defined threshold of 600. Node embeddings $x_{i}\in R^{320}$ were derived from the ESM-2 (8M parameter) model (Lin *et al.* 2023) as larger variants caused severe overfitting. The default edge embedding dimension was 30 (D=10) and varied in ablation studies.

In addition to EGNN (Satorras *et al.* 2021)-based IgPose models, we also implemented two variants and trained them with the SID-CA dataset. First, we substituted EGNN (Satorras *et al.* 2021) with FastEGNN (Zhang *et al.* 2025) and performed weighted sum pooling over all nodes. The second model includes a two-layer high-order equivariant message passing network [MACE (Batatia *et al.* 2022)] followed by a simple sum pooling layer and an MLP classification head.

Our models, implemented in PyTorch and DGL (Wang *et al.* 2019), comprised of four EGNN layers, each followed by a custom GRU gate, with a hidden dimension h=64. The final classifier has an intermediate dropout p=0.1. Training ran up to 50 epochs with early stopping based on validation F1 scores. Adam optimizer was used with an initial learning rate $lr=10^{-4}$ and a cosine annealing scheduler scaling *lr* down to $10^{-5}$. To address class imbalance, a weighted random sampler was used, setting weights for negative and positive samples at 0.8 and 0.2, respectively.

## 3 Results

### 3.1 Classification performance

We evaluated IgPose on three test sets: SID-CA, SID-CB, and CASP-16. For a fair comparison, we ran all baselines on our evaluation set and defined optimal cut-off thresholds based on their respective F-beta scores (Algorithm 2, available as supplementary data at *Bioinformatics* online). We also performed a weighted ensemble of IgPoseClassifier and AbEpiTarget.

**Figure 2:**
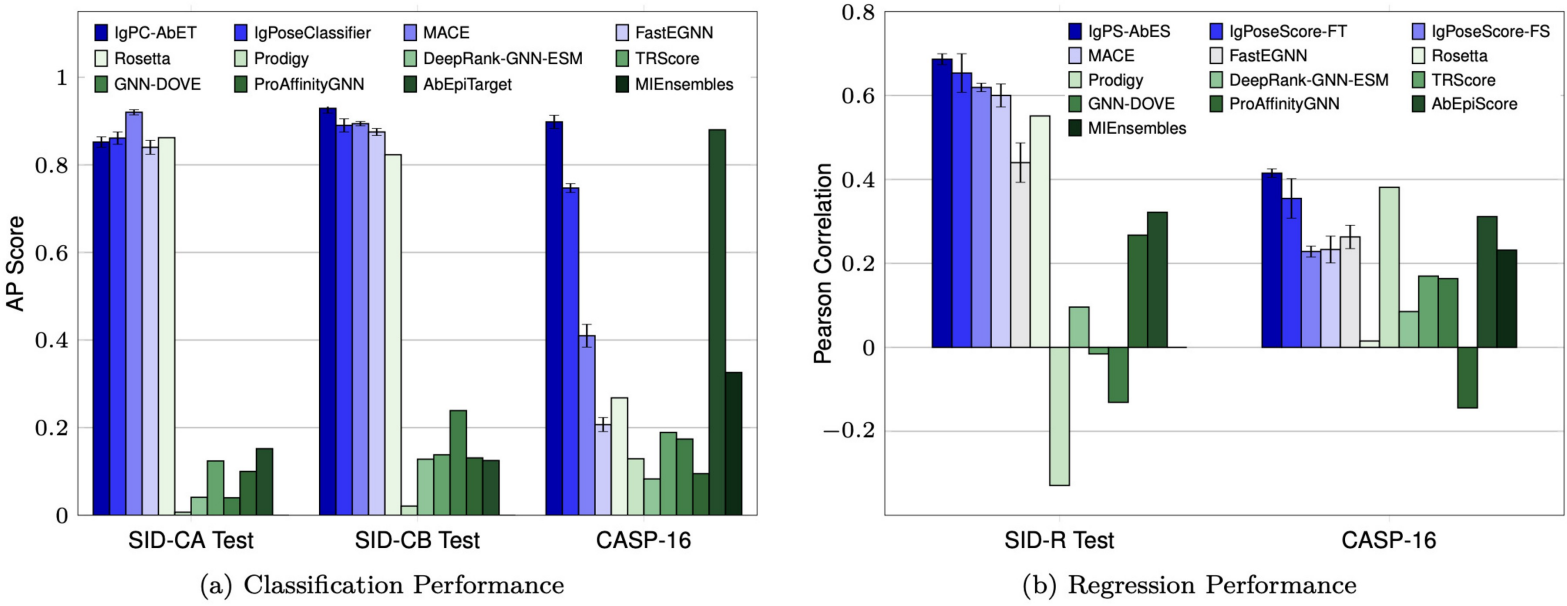
Model performance comparison across (a) classification and (b) regression tasks on our internal SID test datasets and CASP-16. Results of our three implemented models (IgPoseClassifier, FastEGNN, and MACE) are averaged over five executions and colored from pale to deep blue. All existing method results are colored from pale to deep green. In IgPC-AbET and IgPS-AbES settings, we perform weighted average on output probabilities/scores of IgPoseClassifier/IgPoseScore and AbEpiTarget/AbEpiScore with weights of 0.7 and 0.3, respectively. FS and FT refer to two IgPoseScore variants: training from scratch and finetuning from a IgPoseClassifier checkpoint. Error bars of our models represent standard deviation of five executions. Please refer to Table 2 for a detailed comparison of methods on five metrics.

As shown in Fig. 2a and Table 2, IgPose outperforms both physics- and DL-based baselines on AUC and AP scores, showing its strong discriminatory power across all generation protocols: Chai-1 (SID-CA), Boltz-2 (SID-CB), or mixed (CASP-16). Physics-based methods behave inconsistently: Rosetta performs well on SID-C but drops to modest accuracy on CASP-16, while Prodigy underperforms on the SID-C test set and shows only marginal improvement on CASP-16. All general-purpose DL models demonstrate poor performance across the three tasks, suggesting poor generalization. MIEnsembles, a top EMA method from (CASP-16 2024), achieves a slightly better AP score on CASP-16 than both energy-based and general DL-based methods. Interestingly, AbEpiTarget outperforms IgPoseClassifier on CASP-16 despite its limited performance on the SID-C test set, likely due to a bias toward its AlphaFold-Multimer-generated data (Evans *et al.* 2021, Clifford *et al.* 2025). Among our variants, FastEGNN (Zhang *et al.* 2025) and MACE (Batatia *et al.* 2022) only achieve high AUC and AP scores on our internal SID-C tests, while the ensemble of IgPoseClassifier and AbEpiTarget (IgPC-AbET) consistently outperforms all methods.

**Table 2:** Detailed classification performance comparison of our three implemented models (FastEGNN, MACE, IgPoseClassifier) and baselines on two internal test sets and CASP-16 on Precision (P), Recall (R), F1, AUC-ROC (AUC), and AUC-PR (AP) scores.^a^

Method	**SID-CA**					**SID-CB**					**CASP-16**				
	P	R	F1	AUC	AP	P	R	F1	AUC	AP	P	R	F1	AUC	AP
MIEnsembles														0.894	0.326
Prodigy	0.008	**1.000**	0.015	0.071	0.007	0.012	**1.000**	0.024	0.093	0.021	0.080	**1.000**	0.148	0.677	0.129
Rosetta	0.598	0.871	0.709	0.959	0.862	0.435	0.857	0.577	0.939	0.823	0.127	0.983	0.225	0.884	0.268
TRScore	0.249	0.108	0.150	0.623	0.124	0.281	0.113	0.161	0.632	0.138	0.241	0.192	0.214	0.781	0.189
GNN-DOVE	0.040	0.156	0.063	0.380	0.040	0.735	0.136	0.229	0.507	0.239	0.143	0.980	0.250	0.811	0.174
ProAffinityGNN	0.055	0.232	0.089	0.625	0.100	0.092	0.233	0.132	0.785	0.131	0.131	0.418	0.200	0.573	0.095
DR-GNN-ESM	0.037	0.270	0.065	0.414	0.041	0.074	0.263	0.116	0.513	0.128	0.117	0.921	0.208	0.565	0.083
AbEpiTarget	0.293	0.120	0.170	0.705	0.152	0.217	0.135	0.167	0.621	0.125	**0.591**	0.854	**0.699**	**0.965**	0.880
IgPoseClassifier	**0.940**	0.490	0.644	0.982	0.888	**0.967**	0.466	0.628	0.981	0.917	0.474	0.900	0.621	0.914	0.747
IgPC-AbET	**0.940**	0.477	0.633	0.987	0.888	0.964	0.476	0.637	**0.990**	**0.945**	0.568	0.897	0.696	0.928	**0.896**
MACE	0.898	0.895	**0.897**	0.972	**0.920**	0.828	0.873	**0.850**	0.961	0.894	0.176	0.914	0.295	0.888	0.459
FastEGNN	0.923	0.362	0.520	0.982	0.840	0.927	0.343	0.500	0.981	0.875	0.000	0.000	0.000	0.813	0.207

a Baseline methods produce unique results for each dataset, IgPoseClassifier’s results are from its deployed version. In IgPC-AbET setting, we perform weighted average on output probabilities of IgPoseClassifier and AbEpiTarget with weights of 0.7 and 0.3, respectively. Bold and underlined text represent the best and second-best scores of a metric accordingly.

**Figure 3:**
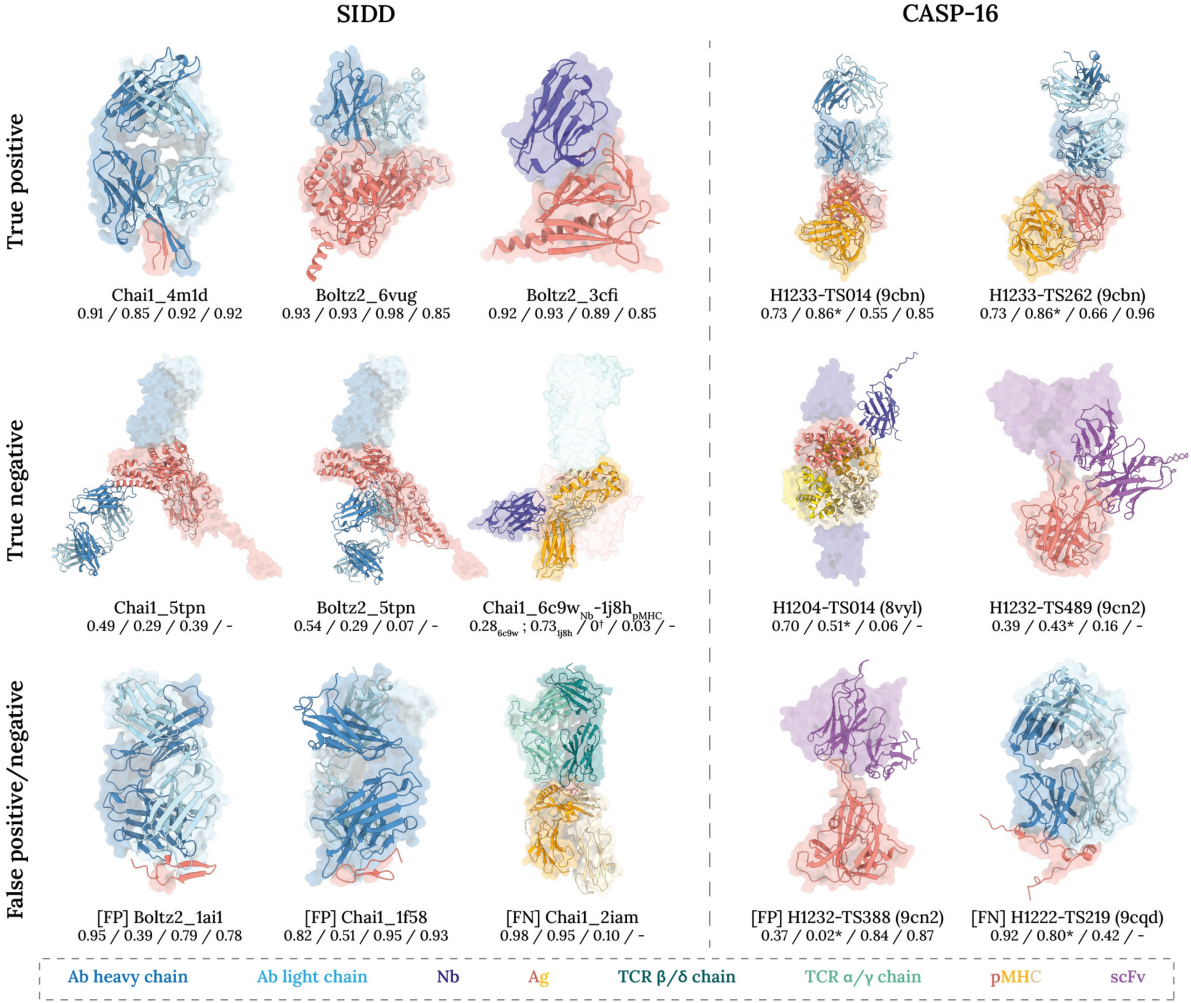
Representative decoy structures from our SIDD test dataset and CASP-16 competition submissions. The decoy structures—shown as cartoon—are categorized into four groups based on the IgPoseClassifier prediction: true positive, true negative, false positive, and false negative. Ground truth structures are shown as transparent surface. For SIDD dataset structures, the prefixes “Chai1” and “Boltz2” specify the computational method used for decoy generation, followed by letters that reference the original PDB-ID. For CASP-16 structures (CASP-16 2024), “H12[number]” denotes the CASP-16 target ID and “TS[number]” corresponds to the participating group that submitted the prediction. PDB-IDs of ground-truth structures are shown in parenthesis. Each structure is accompanied by four numerical scores: (from left to right) global TMscore, global DockQ score, IgPoseClassifier score, and IgPoseScore. IgPoseScore was computed for true/false positive structures only. Asterisk (*) indicates DockQ scores provided by CASP-16 assessors; dagger (†) indicates incomputable DockQ scores, which apply exclusively to non-cognate Ig–Ag decoys. Color-coding scheme used to distinguish antibodies (Ab), nanobodies (Nb), antigens (Ag), T-cell Receptors (TCR), peptide-MHC complexes (pMHC), and single-chain variable fragments (scFv) is indicated at the bottom of the figure.

In Fig. 3, we illustrate representative decoy structures from SIDD and prediction submissions in CASP-16, categorized into TP, TN, FP, and FN according to IgPoseClassifier predictions. These examples encompass structures generated using established structure prediction tools such as Chai-1 (Discovery *et al.* 2024) and Boltz-2 (Passaro *et al.* 2025, Wohlwend *et al.* 2025), along with various approaches used by independent groups in the CASP-16 competition (CASP-16 2024), which collectively capture the structural diversity of binding interfaces, chain compositions, and epitope-paratope arrangements. Each structure is annotated with TM-score (Zhang *et al.* 2022), DockQ (Basu and Wallner 2016), IgPoseClassifier probabilities, and IgPoseScore, enabling quantitative evaluation. These results demonstrate IgPoseClassifier’s ability to identify native-like interfaces, while also highlighting its challenges with specific decoys.

### 3.2 Regression performance

We next evaluated the binding quality scoring capability of IgPoseScore against the baseline methods (Fig. 2b). Each baseline used distinct scoring modalities to rank Ig–Ag poses: estimated TM-score in MIEnsembles (CASP-16 2024), binding energies in Rosetta (Alford *et al.* 2017) and Prodigy (Xue *et al.* 2016), estimated IoU with crystal structures in AbEpiScore (Clifford *et al.* 2025), $pK_{d}$ in ProAffinityGNN (Zhou *et al.* 2024), probabilities in TRScore (Guo *et al.* 2022), GNN-Dove (Wang *et al.* 2021), and $F_{\text{nat}}$ in DeepRank-GNN-ESM (Xu and Bonvin 2024). For IgPoseScore, we tested two variants: FS (trained from scratch) and FT (finetuned from IgPoseClassifier by replacing the head with regression). We also performed a weighted ensemble of IgPoseScore and AbEpiScore (IgPS-AbES).

As shown in Fig. 2b, IgPoseScore exhibits the highest *r* score on the internal SID-R test set (r=0.653), with the fine-tuned version (IgPoseScore-FT) providing additional gains. While MACE performs comparably to IgPoseScore on SID-R (r=0.634), it underperforms on CASP-16 (r=0.233). Rosetta and Prodigy also show inconsistent performance across the two datasets. Specifically, Rosetta strongly correlates with DockQ on SID-R (r=0.551) but shows little to no correlation on CASP-16. Conversely, Prodigy achieves a surprisingly high correlation score (r=0.3812) on CASP-16 but a negative correlation on SID-R. FastEGNN and MIEnsembles demonstrate moderate performance, with FastEGNN obtaining r=0.440 and r=0.263 on CASP-16 and SID-R, respectively, and MIEnsembles achieving r=0.232 on the CASP-16 dataset (*r* for SID-R is not available). Interestingly, most deep learning baselines (DeepRank-GNN-ESM, TRScore, GNN-DOVE, and ProAffinityGNN) show near-zero or negative correlations. Finally, although AbEpiScore is comparable to IgPoseScore on CASP-16 (r=0.3114 versus r=0.355), it falls behind our model on the SID-R test set (r=0.321 versus r=0.653). However, the weighted ensemble of these two models (IgPS-AbES) achieves the highest *r* scores on the two test sets (0.686 on SID-R and 0.415 on CASP-16).

### 3.3 Candidate selection performance

Practical use cases require accurate ranking of predicted binders to prioritize antibody candidates for experimental validation. We evaluated candidate selection performance by first filtering out predicted non-binders using IgPoseClassifier, AbEpiTarget, and their ensemble IgPC-AbET, then ranking the remaining samples with IgPoseScore, AbEpiScore, and their ensemble IgPS-AbES. Top-K success rates (precision@K) were calculated on SID-R and CASP-16 test sets.

**Figure 4:**
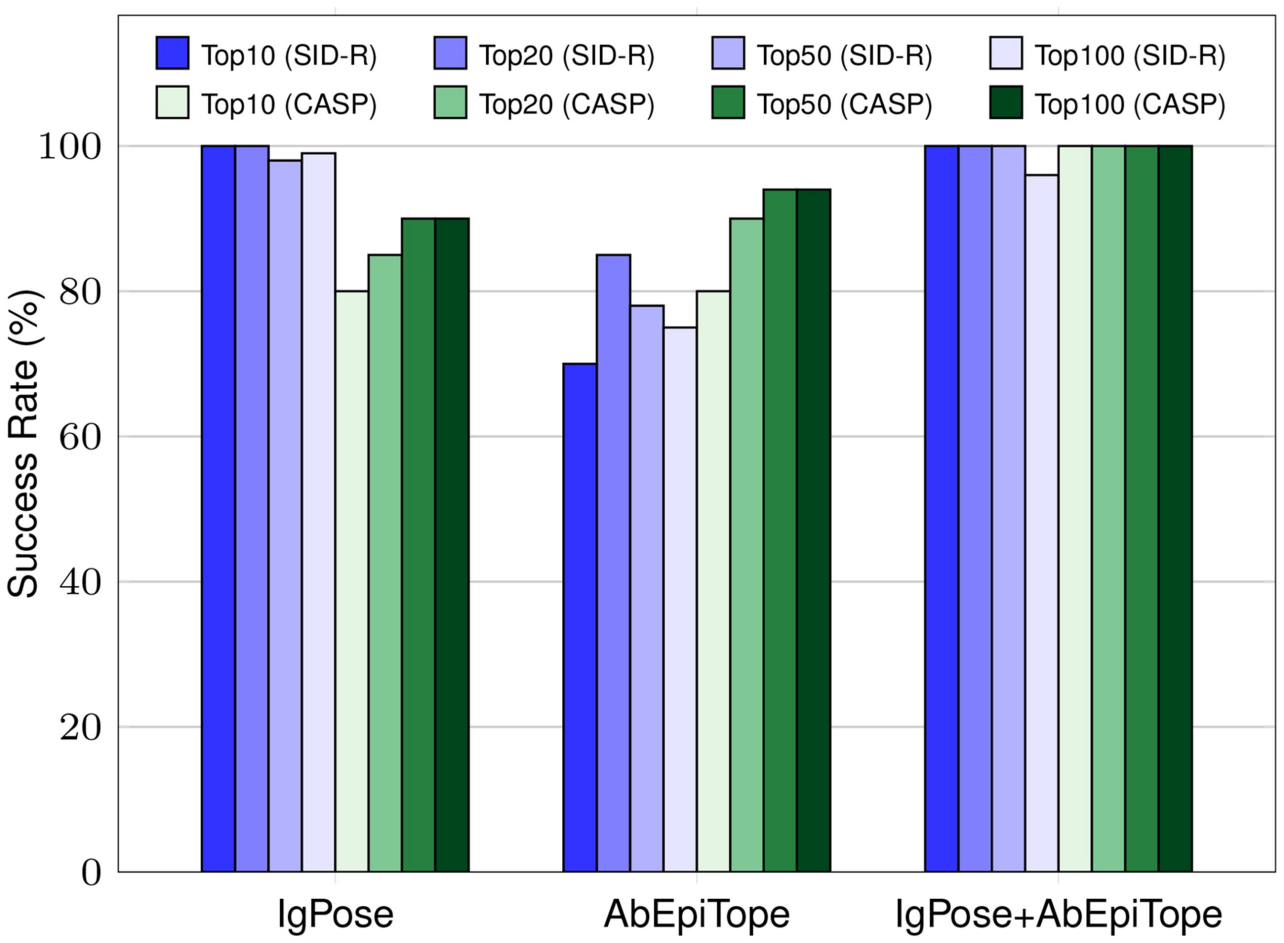
Comparison of Top-K success rates for IgPose and AbEpiTope on two benchmark datasets. The left four (blue-shaded) and the right four (green-shaded) bars show the SID-R and CASP-16 success rates, respectively. Here, success rate is defined as the precision in Top-K: the proportion of true positive samples among the Top-K ranked predictions. Here, IgPoseClassifier or AbEpiTarget are first used to filter out predicted negative samples; the remaining predicted positives are then ranked by IgPoseScore or AbEpiScore; precision is then calculated among the top 10, 20, 50, and 100 ranked candidates. *IgPose+AbEpiTope* denotes the weighted ensemble variant of the two methods.

As shown in Fig. 4, IgPose outperforms AbEpiTope on the SID-R test set, achieving near-perfect success rates: 100% at Top-10 and Top-20, 98% at Top-50, and 99% at Top-100. AbEpiTope-1.0 shows slightly lower precision across all thresholds: 70% at Top-10, 85% at Top-20, 78% at Top-50, and 75% at Top-100. On CASP-16, both models achieve 80% at Top-10. AbEpiTope performs marginally better than IgPose at Top-20, Top-50, and Top-100 (90%, 94%, 94% versus 85%, 90%, 90%). Notably, the ensemble variant outperforms both individual models, achieving nearly perfect scores across all Top-K metrics.

### 3.4 Ablation study

We performed several ablation studies to dissect the contribution of each framework component. The results show that: (i) selective global pooling over broader, non-contacting regions improves robustness on structurally diverse decoys, whereas restricting pooling to CDR/interface regions limits generalization (Fig. S1, available as supplementary data at *Bioinformatics* online), (ii) graph-level augmentations (*k*-hop sampling) stabilize generalization while embedding perturbations degrade it (Fig. S2, available as supplementary data at *Bioinformatics* online), (iii) all-atom graphs clearly outperform $C_{\alpha }$ graphs, and large edge embeddings harm generalization (Fig. S3, available as supplementary data at *Bioinformatics* online), (iv) ensemble methods increase robustness and mitigates overfitting (Fig. S4, available as supplementary data at *Bioinformatics* online), and (v) a customized GRU cell accelerates early learning and yields consistent AP score gains, particularly on CASP-16 (Fig. S5, available as supplementary data at *Bioinformatics* online). Further information can be found in the Supplementary Information.

## 4 Discussion

Modeling Ig–Ag interactions remain challenging, as neither conventional physics-based tools nor DL-based models reliably distinguish true from false binding poses. While general-purpose PPI baselines utilize large-scale training sets such as the Protein–Protein Docking Benchmark (ZDOCK) (Hwang *et al.* 2010, Vreven *et al.* 2015), DockGround (Liu *et al.* 2008, Collins *et al.* 2022), or PDBbind (Wang *et al.* 2004) (with cut-offs typically preceding 2022), they often lack the specialized inductive biases (Battaglia et al. 2018) required for the highly flexible CDR loops found in antibody–antigen interfaces. Furthermore, some general-purpose DL models, such as DeepRank-GNN-ESM (Xu and Bonvin 2024), explicitly exclude antibody data from their training sets. This lack of domain-specific data curation and training likely accounts for their subpar performance on CASP-16 targets compared to the antibody-specific architecture of IgPose. These shortcomings necessitate a unified framework that can filter plausible docking decoys with high precision and estimate Ig–Ag binding quality [e.g. DockQ (Basu and Wallner 2016)] to prioritize candidates for experimental validation.

We developed IgPose, a generative framework that augments limited experimental Ig–Ag complexes with diverse structural decoys generated using Chai-1 (Discovery *et al.* 2024) and Boltz-2 (Passaro *et al.* 2025, Wohlwend *et al.* 2025). IgPose integrates both geometric and evolutionary information to model Ig–Ag binding: it combines equivariant message passing with interface-focused subgraph sampling to capture spatial patterns invariant under physical transformations, while incorporating evolutionary information from ESM-2 protein language model embeddings (Lin *et al.* 2023). IgPose also introduces various pooling strategies to let the neural network prioritize critical information autonomously. Crucially, IgPose provides two complementary tools for evaluating antibody candidates: (i) IgPoseClassifier enables high-confidence discrimination of cognate from non-cognate binding poses, offering an effective filter for large-scale docking pipelines and (ii) IgPoseScore predicts binding pose scores, supporting finer-grained prioritization of antibody candidates.

Empirical evaluations showed that IgPose generalizes effectively across datasets, highlighting the benefits of generative data augmentation and geometry-aware modeling (Fig. 2b). We observed that conventional binding energy estimation tools are significantly more sensitive to the “physicality” of atomic level structural geometries. Rosetta, for instance, likely achieved superior performance on our internal SID test sets by strictly penalizing the unphysical atomic interactions and steric clashes often inherent in hallucinated binding contacts. However, its discriminative power likely diminished on the CASP-16 benchmark due to most submitted structures undergoing extensive conformational refinement (e.g. massive global sampling, relaxation, molecular dynamics refinement), which results in a narrow distribution of Rosetta scores (Fig. S7, available as supplementary data at *Bioinformatics* online), making it difficult to distinguish between a near-native low-energy pose and an energetically minimized but incorrect decoy. In contrast, Prodigy assigns favorable (more negative) binding affinities to structures with a high number of interfacial contacts. This heuristic makes it susceptible to hallucinated interactions in decoys, resulting in an inverse predictive pattern in our internal tests. On CASP-16, Prodigy achieved moderate AUC and *r* scores, reflecting its capacity to distinguish tight interfaces from loose contacts. However, it failed to discriminate high-quality complexes (DockQ≥0.8) from acceptable- and medium-quality poses, resulting in a low AP score. General DL-based prediction models also suffered from out-of-distribution problems, leading to inferior performances across benchmarks. These findings suggest that existing tools remain unreliable for ranking in-silico Ig–Ag structures. Notably, AbEpiTope (Clifford *et al.* 2025), a recent Ab-Ag binding prediction framework, achieved substantial performance on CASP-16 but performed poorly on our internal SID dataset. This performance gap suggests that the model’s efficacy may be sensitive to specific data augmentation strategies and training objectives, and integrating such approaches into our framework is a promising avenue for future investigation.

The Ig–Ag binding prediction field currently lacks standardized, large-scale benchmarks. While the CASP challenge (CASP-16 2024) provides an invaluable external evaluation set, it is constrained by a limited number of cognate Ig–Ag pairs (*n* = 8). The lack of highly accurate Ig–Ag structure modeling tools and limited benchmarks hinder objective comparisons between studies. Although our generative data pipeline partially addresses this scarcity, the curated dataset may introduce a bias toward antibody structures encountered during training, potentially impeding generalization through partial memorization. To mitigate this, we used an ensemble approach (Lakshminarayanan *et al.* 2017). We observed that a simple average of multiple trained checkpoints consistently enhanced IgPose’s performance across all benchmarks. Furthermore, a weighted ensemble of IgPose and AbEpiTope outperformed all comparison methods, further confirming the effectiveness of this synergistic approach. Interestingly, our evaluation of pooling strategies suggests that the model does not prioritize the binding interface in its readout functions, despite this region containing the most biologically informative signals. This discrepancy may arise from data imbalance or inductive biases (Battaglia *et al.* 2018) in architecture, necessitating further investigation into the spatial distribution of attention during training.

From a practical perspective, IgPose is well-suited for therapeutic antibody discovery. In a typical virtual screening workflow, IgPoseClassifier can rapidly discard non-cognate Ig–Ag structures, while IgPoseScore facilitates candidate prioritization through pose scoring. This two-step inference framework optimizes resource allocation for downstream wet-lab validation, particularly when screening against structurally novel antigens or expansive antibody libraries. However, several challenges remain: (i) IgPose’s Pearson correlation with DockQ remains moderate, and (ii) while IgPose demonstrates high accuracy for antibody-antigen binding prediction, we observed comparatively lower performance on the TCR-pMHC subset (Fig. S8, available as supplementary data at *Bioinformatics* online). This disparity highlights the distinct biophysical challenges of TCR recognition, such as the lower binding affinity ranges and more constrained binding orientations compared to antibodies, which will be the focus of future model refinements.

## 5 Conclusion

Existing methods for Ig–Ag pose discrimination and scoring frequently fail to generalize across diverse antigen landscapes. To address this, we developed IgPose, a unified framework designed to enhance the robustness and generalizability of Ig–Ag pose classification and scoring. IgPose achieves this by synergistically integrating generative decoy augmentation, evolutionary context via ESM-2 embeddings, and geometric inductive biases through equivariant graph neural networks (EGNNs) with gated updates. Furthermore, the inclusion of auxiliary losses strategically aligns structural uncertainty with predictive confidence.

Our framework incorporates IgPoseClassifier for identifying near-native cognate poses and IgPoseScore for scoring poses. Compared to traditional physics- and DL-based tools, the two functions of IgPose demonstrated superior classification accuracy and regression correlation, showcasing IgPose’s consistent generalization to unseen structures. Additionally, detailed ablation studies confirmed that interface-focused *k*-hop subgraph sampling and selective global pooling over specific regions are pivotal to the performance of the model. These specific designs align the model’s inductive biases with the complex topology of Ig–Ag structures and interfaces, significantly enhancing discriminative power. We envision IgPose as a practical tool for therapeutic antibody discovery pipelines, where fast and accurate screening of binding poses and affinities can significantly accelerate candidate selection.

## Supplementary Material

btag076_Supplementary_Data

## Data Availability

The Ig–Ag structures used in this study were obtained from public repositories: SAbDab (Dunbar *et al.* 2014), STCRDab (Leem *et al.* 2018), and CASP-16 (CASP-16 2024). The data underlying this article are available on Zenodo (https://doi.org/10.5281/zenodo.17431183).

## Code availability

The source code for IgPose is available on Zenodo (https://doi.org/10.5281/zenodo.17431131) and Github (https://github.com/arontier/igpose).
