# Supplementary material for: IgPose: a generative data-augmented pipeline for robust immunoglobulin–antigen binding prediction
Source: Bioinformatics. 2026 Feb 15;42(3):btag076. doi: 10.1093/bioinformatics/btag076 (PMC12989135; doi:10.1093/bioinformatics/btag076)
Supplement: btag076_Supplementary_Data [file btag076_supplementary_data.pdf]

Supplementary Information for:

IgPose: A Generative Data-Augmented Pipeline for Robust Immunoglobulin-Antigen Binding Prediction

Tien-Cuong Bui[1,†], Injae Chung[1,†], Wonjun Lee[1], Junsu Ko[1,*], and Juyong Lee[1,2,*]

[1]Arontier Co., Ltd., Seoul, 06735, Republic of Korea
[2]Seoul National University, Seoul, 08826, Republic of Korea

[†]These authors contributed equally to this work.
[*]Correspondence: nicole23@snu.ac.kr, junsuko@arontier.co

Additional Experiments

Pooling Strategies

This section describes global pooling strategies presented in **Figure S1**. A detailed comparison is shown in **Table S1**.

- **Pooling over all nodes**: The default configuration in our architecture for the global pooling operator is to perform weighted sum over all nodes in an input graph.
- **Pooling over interface (interface-only)**: Nodes located at the interface region are selected for global pooling operator.
- **Pooling over CDR (CDR-only)**: Nodes located at the CDR region are selected for global pooling operator.
- **Pooling over CDR-Epitope (CDR-Epitope-only)**: Nodes located at the CDR and epitope regions are selected for global pooling operator.
- **Excluding interface (w/o interface)**: Nodes located at the interface region are selected for global pooling operator.
- **Excluding CDR (w/o CDR)**: Nodes located at the CDR region are selected for global pooling operator.
- **Excluding CDR-Epitope (w/o CDR-Epitope)**: Nodes located at the CDR and epitope regions are selected for global pooling operator.
- **Ensemble of 3 best**: Predicted probabilities of three best pooling techniques are averaged and selected as the final predictions.

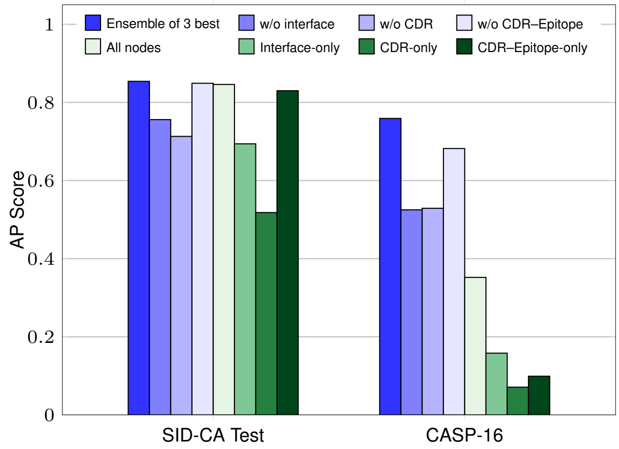

Fig. S1. A comparison of performance of pooling strategies on SID-CA test and CASP-16 benchmarks. Labels denote the set of nodes used in the global weighted sum pooling operation. 'w/o' denotes exclusion of the specified node set from pooling, while the suffix 'only' indicate exclusive use of the specified set of nodes. All node: weighted sum pooling over all nodes in a graph. Ensemble of 3 best: average of output probabilities from the three models corresponding to the pale blue bars. Further detail can be found in **Section S1.1**.

Selective Global Pooling Strategies

To determine the structural information essential for accurate predictions, we evaluated the impact of global pooling operations on model performance. We employed two strategies: one that excludes the Ig-Ag interface, CDR region, and CDR-epitope interface, and another that used only these specific regions. We also assessed two additional configurations: an ensemble that averaged predictions from the three best models and a baseline that applied weighted sum pooling across all nodes.

Pooling strategies yield similarly high AP scores on SID-CA, suggesting that the choice of pooling region has limited impact on this curated internal dataset (**Figure S1**), which may be due to partial memorization of structural patterns during training. The CASP-16 set [CASP-16, 2024] inherently has a greater structural diversity reflecting a broad spectrum of computational prediction and refinement protocols employed by various participants. On this unseen dataset, broader pooling strategies that incorporate non-contacting scaffold regions significantly enhance model robustness by capturing the global topological features of the Ig–Ag complex. Specifically, pooling strategies that focus on the framework while excluding the immediate interface or CDR loops performed substantially better than global pooling over all nodes. This suggests that non-contacting regions of the immunoglobulin and antigen provide a critical geometric reference frame, effectively 'anchoring' the binding site within the overall three-dimensional structure. Conversely, restricted pooling over only the CDR or CDR–epitope regions leads to a sharp degradation in performance, demonstrating that the hypervariable CDR loops alone lack sufficient geometric or contextual information for out-of-distribution generalization. Their high conformational plasticity and sequence variability likely render local-only signals too 'noisy' to reliably rank poses without the stabilizing context provided by the conserved protein scaffold.

Data Augmentation Methods

Here, we describe the data augmentation methods presented in **Figure S2**. A detailed comparison is shown in **Table S1**. The sampling algorithm is described in **Algorithm 1**.

- **Nondocking**: $\mathcal{E}_{\text{inter}}$ is discarded from computational graphs when input to models. In this setting, no sampling method is applied to graphs.
- **Random rotation**: During training, positions of nodes in input graphs are rotated by random angles.

- **Mask embed**: Residues of nodes excluded from the sampling process are masked out from the residue sequence before performing ESM-2.
- **Mask embed + random rotation**: First random rotation is applied to graphs in training. Then, residue characters of unselected nodes in a computational graph are masked out from the input sequence before the embedding generation step.
- **3-hop sampling with node threshold (3-hop interface)**: All nodes included in inter-Ig-Ag edges are selected as seed nodes. A Breadth First Search (BFS) sampling procedure starts picking nodes layer by layer. The process stops when the number of selected nodes exceeds a pre-defined threshold.
- **CDR sampling with node threshold (3-hop CDR)**: Similar to the strategy above but seed nodes are only those located at the CDR region.

This experiment investigated whether graph-level augmentation strategies improve model robustness under distribution shifts. We grouped augmentations into three main categories: (i) graph sampling based on k-hop iterations with interface or CDR anchors; (ii) geometric perturbations with random rotations in training; and (iii) node embedding modifications with sequence masking before ESM-2 execution. For a detailed view of their results on the five metrics, please refer to **Table S2**.

As shown in **Figure S2** and **Table S2**, the 3-hop interface method achieved the highest AP score (0.846) on the SID-CA test set. Although all methods suffer performance drop on CASP-16, the 3-hop interface sampling approach remained the most resilient, maintaining the highest AUC (0.891) and AP (0.352) scores. Methods that applied additional geometric changes or embedding-level noise, such as 'Random rotation', 'Mask Embed' and 'Mask Embed + random rotation', saw a drastic decline in performance on CASP-16, suggesting that perturbing coordinates or features is insufficient for cross-dataset generalization. Moreover, the 'Nondocking' approach achieved performance comparable to the CDR-based sampling method, suggesting that the model likely learned shapes from individual protein graphs rather than relying solely on explicit interaction edges.

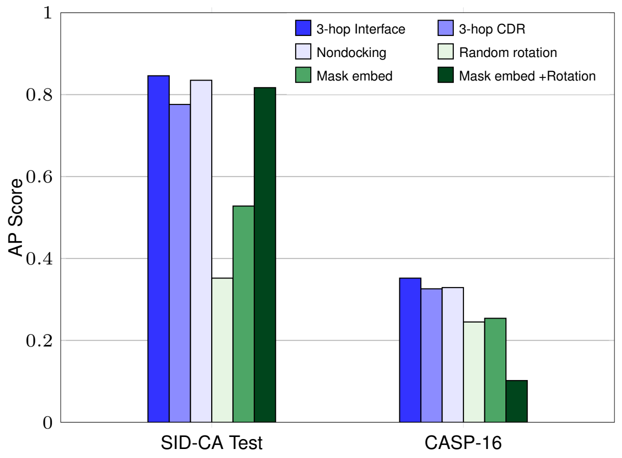

Fig. S2. Performance comparison of different data augmentation strategies on CASP-16 benchmark in AP score. Here, 'interface' and 'CDR' mean the selected seed sets for initializing the sampling procedure. 'Nondocking' is the setting, where inter Ig-Ag edges are removed from $\mathcal{G}$. For a detailed description of augmentation strategies, please refer to Section S1.3. '3-hop' refers to a 3-hop BFS sampling procedure starting from a set of seed nodes and finishing once reaching a node threshold (600). All models perform weighted sum over all nodes in graphs.

Graph Construction Methods

To assess the impact of graph topology and edge-embedding sizes on CASP-16 [CASP-16, 2024] performance, we compared various graph construction schemes. The all-atom (AA) strategy consistently

Table S1. Detailed classification performance comparison of pooling strategies on SID-CA test and CASP-16 on Precision (P), Recall (R), F1, AUC-ROC (AUC), and AUC-PR (AP) scores. Bold and underlined text represent the best and second best scores of a metric accordingly. Best checkpoints are selected based on AP scores on the evaluation set.

Strategy	Our Test Set					CASP-16				
	P	R	F1	AUC	AP	P	R	F1	AUC	AP
Pooling over all nodes	0.866	0.723	**0.788**	<u>0.968</u>	0.846	0.353	<u>0.900</u>	0.507	0.891	0.352
w/o interface nodes	0.627	**0.868**	0.728	0.959	0.756	0.157	**0.908**	0.268	0.902	0.525
w/o CDR nodes	0.598	<u>0.824</u>	0.693	0.959	0.713	0.192	**0.908**	0.317	**0.916**	0.529
w/o CDR-Epitope nodes	0.954	0.334	0.494	0.966	0.849	**0.653**	0.868	**0.745**	0.910	0.682
interface-only nodes	0.675	0.802	0.733	0.958	0.694	0.181	0.894	0.301	0.776	0.158
CDR-only nodes	0.814	0.255	0.388	0.899	0.518	0.001	0.003	0.002	0.462	0.071
CDR-Epitope-only nodes	0.901	0.671	<u>0.769</u>	0.955	0.830	0.000	0.000	0.000	0.639	0.099
Ensemble of 3 best checkpoints	<u>0.932</u>	0.502	0.653	0.967	<u>0.854</u>	<u>0.400</u>	0.897	<u>0.553</u>	<u>0.915</u>	**0.759**
Ensemble of 5 best checkpoints	**0.934**	0.519	0.668	**0.969**	**0.869**	0.392	<u>0.900</u>	0.546	<u>0.915</u>	<u>0.755</u>

Table S2. Detailed classification performance comparison of data augmentation methods on SID-CA test and CASP-16 on Precision (P), Recall (R), F1, AUC-ROC (AUC), and AUC-PR (AP) scores. Weighted sum pooling over all nodes are applied to all settings. Bold and underlined text represent the best and second best scores of a metric accordingly.

Method	Our Test Set					CASP-16				
	P	R	F1	AUC	AP	P	R	F1	AUC	AP
Nondocking	**0.920**	0.586	<u>0.716</u>	**0.973**	<u>0.835</u>	0.171	**0.911**	0.288	0.875	<u>0.329</u>
Random rotation	0.457	<u>0.781</u>	0.576	0.937	0.352	0.156	<u>0.908</u>	0.266	0.854	0.245
Mask Embed	0.617	0.529	0.570	0.945	0.528	<u>0.338</u>	0.897	<u>0.491</u>	0.858	0.254
Mask Embed + random rotation	0.800	0.516	0.628	**0.973**	0.817	0.132	0.622	0.218	0.649	0.102
3-hop sampling with node threshold	<u>0.866</u>	0.723	**0.788**	<u>0.968</u>	**0.846**	**0.353**	0.900	**0.507**	**0.891**	**0.352**
CDR sampling with node threshold	0.601	**0.828**	0.696	0.957	0.776	0.168	<u>0.908</u>	0.284	<u>0.883</u>	0.326

outperformed the C_α-based method in AUC and AP (**Figure S3**), indicating that side-chain and backbone atoms provide important geometric information.

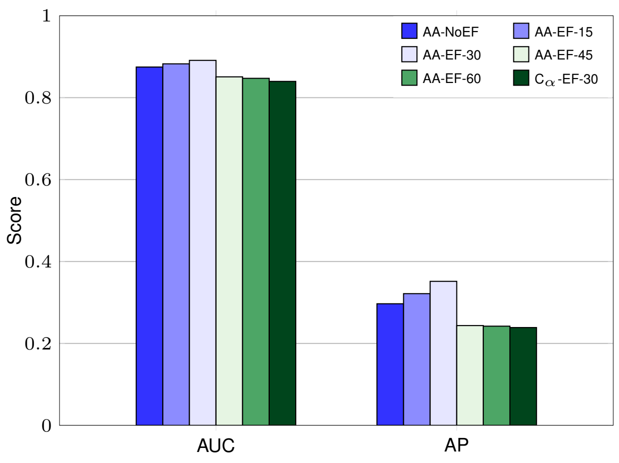

Fig. S3. Performance comparison across graph construction methods on CASP-16. Here, AA (all-atom) denotes the baseline setting, C_α means edges in a graph are established based on C_α distances, and EF represents edge features with a corresponding size. All models perform weighted sum global pooling over all nodes in graphs.

In AA models, small edge embedding dimensions (0-30) had no discernible impact on model performances, likely because the EGNN architecture inherently encodes distance information within its radial network (**Figure S3**). However, larger embedding sizes (45 and 60) substantially degraded model performance, resulting in an approximate 10-point drop in AP score. These results highlight the critical need to explore alternative strategies for generating edge attributes to improve model generalization.

Overfitting and generalization challenges

Given the complex contextual nature of Ig-Ag, the distribution shift problem is inevitable. We performed inferences on the SID-CA test set and CASP-16 [CASP-16, 2024] and visualized their probability distributions. The two datasets show different trends in probability distribution as the training process progresses: the SID-CA test set's probabilities gradually shift to the left and have a long right tail reflecting the imbalance of positive samples in the training set, while the CASP-16 dataset's probabilities shift to the right indicating model bias toward positive samples (**Figure S4**). Furthermore, the AP score in the SID-CA test set increases steadily from Epochs 1 to 5 and remains stable, while this score in the CASP-16 benchmark degrades as training progresses (**Figure S5**). These results indicate that there is a degree of overfitting in the current training paradigm, especially when an oversampling method is used to increase the occurrence of positive samples. Practically, we can apply ensemble techniques to aggregate predictions of different models to alleviate this problem by leveraging the diversity of individual models to create a more robust and generalized prediction.

Customized GRU for Learning Acceleration

We customized the original GRU cell [Cho et al., 2014b] by adding an additional connection between the previous hidden state and the current input. Practically, this modification accelerates model learning. As shown in **Figure S5**, AP scores of the customized version are higher than those of the original version in the first

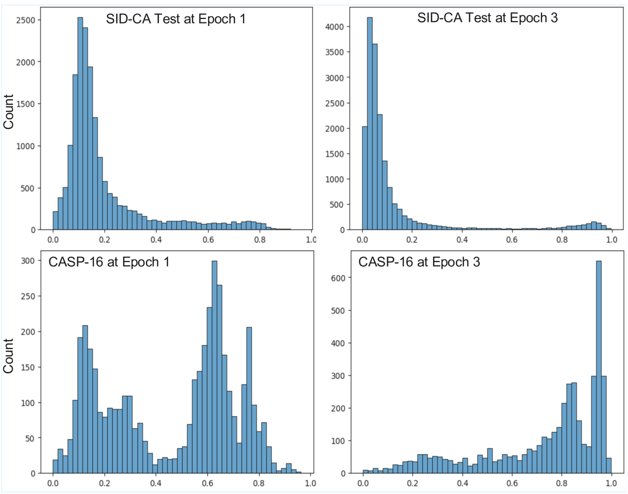

Fig. S4. Change of probability distributions over three epochs for SID-CA test set (top) and CASP-16 (bottom). Probability distributions of the two test datasets change differently as training progresses.

epoch on both datasets, especially on CASP-16 [CASP-16, 2024]. The trend in performance gain continues until Epoch 5 on our test set. These results indicate that the additional connection in GRU enables models to learn quickly from the training data.

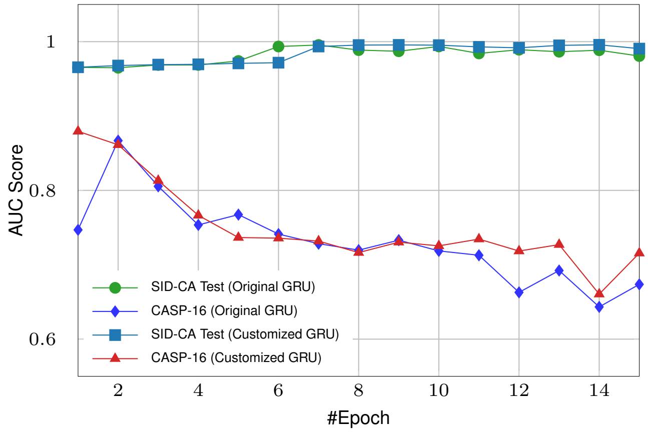

Fig. S5. AUC Score trends over 15 training epochs for IgPoseClassifier's GRU variants on SID-CA test set and CASP-16. All models use the all-node global pooling operator in this experiment.

Performance of Top EMA Methods on CASP-16

In the Quality Assessment (QA) category of CASP-16 [CASP-16, 2024], participants do not predict structures; rather, they evaluate and rank ensembles of decoys generated by other prediction servers. To benchmark IgPose against these state-of-the-art Estimation of Model Accuracy (EMA) methods, we retrieved all submissions corresponding to our eight CASP-16 structural targets. We then extracted both the overall score and the interface scores from 'QMODE 1' in all submissions. Due to missing interface scores in several entries, we evaluated performance using AUC, AP, and Pearson correlation (r) based on overall scores and DockQ. For clarity, we report only the top four performing EMA methods—prioritized by Area Under the Precision-Recall Curve

(AP)—as the remaining entries yielded AP scores below 0.1. As illustrated in **Figure S6**, the predictive performance of current EMA methods remains limited on Ig–Ag targets, particularly in terms of AP and r scores. These results underscore the inherent difficulty of the antibody–antigen binding prediction problem and suggest that general-purpose QA methods struggle to capture the specific biophysical nuances of immunoglobulin interfaces.

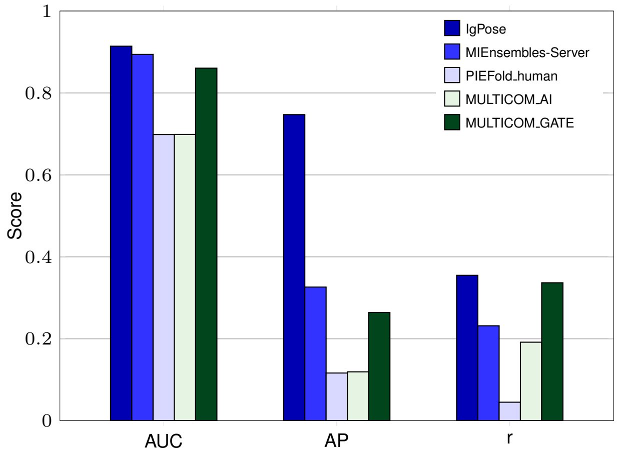

Fig. S6. Performance of IgPose and top EMA methods reported in CASP-16 [CASP-16, 2024]. We downloaded predicted results from CASP-16 and computed AUC, AP, and r scores.

Analysis of Score Distributions of Rosetta and Prodigy

We investigated the discrepancies in the predictive performance of physics-based energy estimation baselines, specifically Rosetta [Alford et al., 2017] and Prodigy [Xue et al., 2016] (**Figure S7**). For Rosetta, the CASP-16 structures exhibited a significantly narrower score distribution concentrated at highly favorable (low) binding energies compared to the broader distribution observed in the SID-CA dataset. We attribute this to the fact that CASP-16 submissions typically undergo extensive conformational refinement and energy minimization. This process effectively flattens the local energy landscape, diminishing Rosetta's discriminative power as both near-native and incorrect poses occupy a similar low-energy range. This phenomenon explains the decline in Rosetta's classification performance on the CASP-16 benchmark. In contrast, Prodigy exhibited an inverse predictive pattern on the SID-CA dataset (AUC $= 0.071$; **Table 2**). This is likely because Prodigy relies on a contact-counting heuristic, causing synthetic decoys with high-density, hallucinated interfacial contacts to receive more favorable binding affinity scores than the true native poses. On CASP-16, Prodigy achieved moderate performance (AUC $= 0.677$, $r = 0.381$, AP $= 0.129$). This suggests that while Prodigy can differentiate between tight and loose interfacial packing, it lacks the geometric sensitivity required to distinguish high-quality complexes (DockQ ≥ 0.8) from 'acceptable' to 'medium' quality decoys [Basu and Wallner, 2016].

Classification Performance on Ab-Ag, Nb-Ag, TCR-pMHC subsets

We evaluated the performance of IgPoseClassifier across the Ab-Ag, Nb-Ag, and TCR-pMHC subsets, with dataset statistics (**Table S3**) and classification metrics (**Figure S8**).

As shown in **Figure S8**, IgPose demonstrates high predictive accuracy for Ab-Ag and Nb-Ag subsets, but encounters specific challenges when evaluating TCR-pMHC binding interactions. While AUC and AP scores remain high for Ab-Ag and Nb-Ag, the lower evaluation scores for TCR-pMHC suggest that this subset is more difficult for the current architecture to characterize accurately. The difference in performance is likely influenced by the unique biophysical properties of TCR-pMHC pairings, which are often of lower affinity and structurally more rigid compared to the more flexible Ab-Ag interactions. Furthermore, we observed an extreme data imbalance within the TCR subset, which contains the smallest ratio of positive samples (**Table S3**), presenting a huge challenge for IgPose in learning distinctive geometric patterns. These results suggest two potential directions for improving IgPose's performance on TCR-pMHC subset: (i) investigating the geometric differences between TCR-pMHC and Ab/Nb-Ag binding interfaces, and (ii) addressing the severe data imbalance in this subset.

Table S3. Dataset Statistics for Ab-Ag, Nb-Ag, TCR-pMHC Subsets in SID-CA and SID-CB.

Type	SID-CA		SID-CB	
	#pos	#neg	#pos	#neg
Ab-Ag	701	679	738	153
Nb-Ag	198	14,230	228	14,126
TCR-pMHC	27	2,139	35	2,157

Algorithms

This section presents fundamental algorithms embedded in the IgPose architecture.

Theoretical Analysis of Global Pooling and Information Propagation

To interpret the ablation results regarding the read-out set $\mathcal{S}$, we analyze the information flow within the network. Let the input graph be $\mathcal{G} = (\mathcal{V}, \mathcal{E}, \mathcal{X}_v, \mathcal{X}_e, \mathcal{P})$, where $\mathcal{V}$ is the set of residues. The network depth is defined by T layers.

As described in **Eq. 2**, the node features $H^{(t)}$ at layer t are updated via an EGNN layer followed by a customized GRU gated update. We can rewrite **Eq. 2** in the node-level format as follows:

$$\begin{aligned} \tilde{h}_i^{(t)}, p_i^{(t)} &= \text{EGNN}\left(h_i^{(t-1)}, p_i^{(t-1)}, \{h_j^{(t-1)}, p_j^{(t-1)}\}_{j\in\mathcal{N}(i)}\right), \\ h_i^{(t)} &= \text{GRU}\left(\left[\tilde{h}_i^{(t)}, h_i^{(t-1)}\right], h_i^{(t-1)}\right) \end{aligned} \tag{11}$$

where i and j are node indices, and $\mathcal{N}_i$ denotes a set of neighbors of the node i.

Eq. 11 establishes that $h_i^{(t)}$ is a function of the local neighborhood at $t-1$. We define the structural receptive field $\mathcal{R}_i^{(t)}$ of node i at the layer t as the set of input nodes that influence its state:

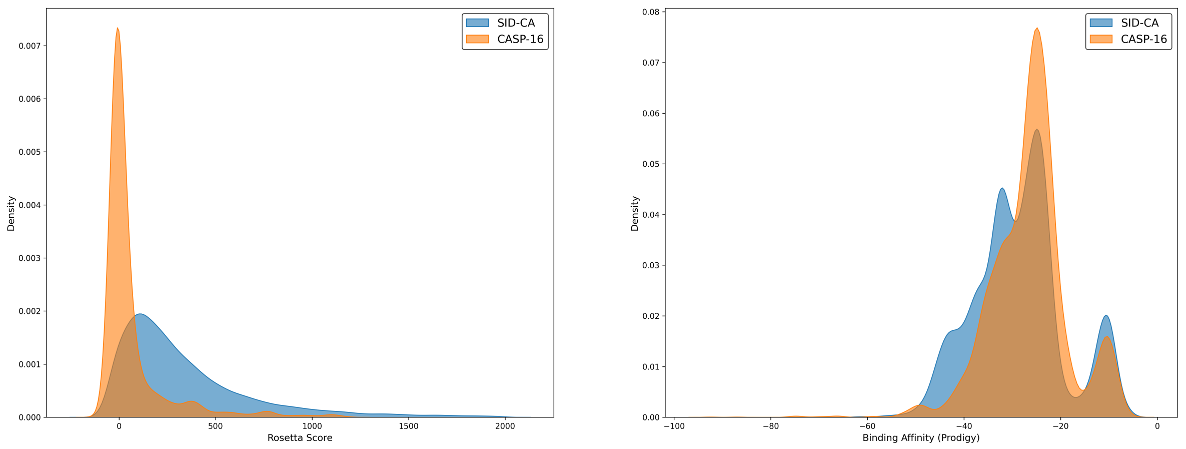

(a) Rosetta Binding Energy (b) Prodigy Binding Affinity

Fig. S7. Density distributions of Rosetta binding energy and Prodigy binding affinity scores. CASP-16's structures show narrower distributions concentrated at lower energy values compared to SID-CA, reducing the discriminative power of energy-based scoring functions.

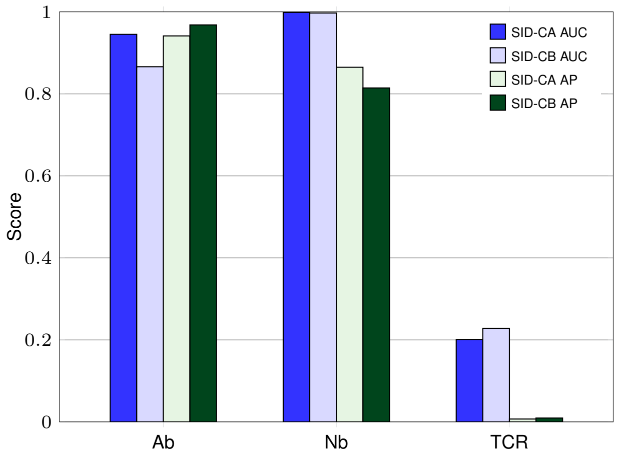

Fig. S8. Performance comparison on Ab-Ag, Nb-Ag, and TCR-pMHC subsets.

$$\mathcal{R}_i^{(0)} = i, \quad \mathcal{R}_i^{(t)} = \mathcal{R}_i^{(t-1)} \cup \bigcup_{j \in \mathcal{N}(i)} \mathcal{R}_j^{(t-1)}. \tag{12}$$

In other words, a node i's state at the layer t depends on every node that directly influenced i and other nodes that affected each of its neighbors at the previous layer $t-1$.

Given T layers, the final embedding $h_i^{(T)}$ aggregates information from the T-hop neighborhood of node i. Since the protein graph is connected via peptide bonds and inter-residue contacts, for a sufficient T, the receptive field of a framework node v_{frame} expands to include interface nodes v_{int}. Thus, the final embedding $h^{(T)}$ of a non-interface node i is conditionally dependent on the state of interface nodes:

$$h_i^{(T)} = f\left(\left\{h_k^{(0)} : k \in \mathcal{R}_i^{(T)}\right\}\right) \tag{13}$$

The global graph embedding g is computed via a weighted sum over a selected subset $\mathcal{S} \subset \mathcal{V}$:

$$g(\mathcal{S}) = \sum_{i \in \mathcal{S}} \sigma(w_p^\top h_i^{(T)} + b_p) \odot h_i^{(T)}. \tag{14}$$

Specifying a subset $\mathcal{S}$ that maximizes classification performance (perturbation method) is prevalent in interpretable GNNs [Bui et al., 2023, Bui and Li, 2023]. The exploration procedure can be guided by either learning algorithms or domain expertise. Given the large scale of computational protein graphs, we opt for the latter approach and left the first one for future exploration. Our empirical observations demonstrate that defining $\mathcal{S}$ as the set of non-interface (or non-CDR-epitope) nodes maximizes discriminative power (high AP & AUC scores). This phenomenon can be referred to as the inductive bias of message-passing networks in decoy discrimination tasks.

While the interface region ($\mathcal{V}_{\text{int}}$) contains the direct binding contacts, it is also the region of highest variance and noise in generated decoys (e.g., local steric clashes or side-chain overlap). Conversely, the connecting framework nodes ($\mathcal{V}_{\text{frame}}$) can act as specific "sensors" that integrate these local perturbations. A "bad" interface induces gradient updates that propagate to the framework, manifesting as geometric strain or latent feature inconsistency in the surrounding nodes.

Therefore, by performing weighted sum pooling over $\mathcal{S} = \mathcal{V} \setminus \mathcal{V}_{\text{int}}$, the readout function focuses on the propagated structural consistency of the complex rather than the noisy local features of the contact boundary. The model learns to discriminate true poses versus wrong ones through indirect signals passed to "scaffold" regions.

Mathematical Justification for Modified GRU

The standard GRU Cho et al. [2014b] updates the hidden state $H^{(t-1)}$ through a gating mechanism. Formally, the reset gate $r^{(t)}$ and update gate $z^{(t)}$ are computed as:

$$\begin{aligned} r^{(t)} &= \sigma\left(W_r^{(i)} \widetilde{H}^{(t)} + W_r^{(h)} H^{(t-1)} + b_r\right) \\ z^{(t)} &= \sigma\left(W_r^{(i)} \widetilde{H}^{(t)} + W_r^{(h)} H^{(t-1)} + b_z\right) \end{aligned} \tag{15}$$

In the candidate hidden state $n^{(t)}$, the reset state $r^{(t)}$ multiplies to $H^{(t-1)}$ in element-wise, strictly controlling the historical context used for the new proposal:

$$n^{(t)} = \tanh\left(W_n^{(i)}\widetilde{H}^{(t)} + W_n^{(h)}(r^{(t)} \odot H^{(t-1)}) + b_n\right) \tag{16}$$

The final hidden state $H^{(t)}$ is a linear interpolation between the previous state and the candidate state:

$$H^{(t)} = (1 - z^{(t)}) \odot n^{(t)} + z^{(t)} \odot H^{(t-1)} \tag{17}$$

We modify the GRU function by defining an input vector $X^{(t)} = \left[\widetilde{H}^{(t)}, H^{(t-1)}\right]$. By expanding the update gate $z^{(t)}$ (or the reset gate $r^{(t)}$), we can group terms as follows:

$$\begin{aligned} z^{(t)} &= \sigma\left(W_z^{(i)}X^{(t)} + W_z^{(h)}H^{(t-1)} + b_z\right) \\ &= \sigma\left(W_z^{(i,\widetilde{H})}\widetilde{H}^{(t)} + \mathbf{W_z^{(i,H)}H^{(t-1)}} + W_z^{(h)}H^{(t-1)} + b_z\right) \\ &= \sigma\left(W_z(i,\widetilde{H})\widetilde{H}^{(t)} + (\mathbf{W_z^{(i,H)}} + W_z^{(h)})H^{(t-1)} + b_z\right) \end{aligned} \tag{18}$$

As can be seen, we are now having two independent weights operating on $H^{(t-1)}$. If we use Xavier or Kaiming method for weight initialization, these operations can increase pre-activation variance and saturation of r and z gates. Specifically, increasing the magnitude of pre-activation via the double-weight transformation pushes the gate values away from 0.5 toward the saturation regions (0 or 1) as the Sigmoid function is most sensitive in range $[-2, 2]$. Furthermore, r and z gates are more sensitive to $H^{(t-1)}$ as a node is listening to its own history twice as loudly as it is listening to the neighbors.

In standard GNN architectures, node representations tend to become homogeneous across nodes (oversmoothing) as the number of layers increases. The modified GRU enforces a strong self-loop for each node. The candidate state $n^{(t)}$ relies not only on the neighbor-averaged signal $\widetilde{H}^{(t)}$ but also directly on the node's previous features via $W_i^{(n,H)}$. This additional linear transformation allows the model to selectively balance graph topology information with temporal changes, resulting in more stable performance for dynamic physical systems. The modified candidate hidden state $n^{(t)}$ is as follows:

$$\begin{aligned} n^{(t)} &= \tanh\left(W_i^{(n)}X^{(t)} + W_h^{(n)}(r^{(t)} \odot H^{(t-1)}) + b_n\right) \\ &= \tanh\Big(W_i^{(n,\widetilde{H})}\widetilde{H}^{(t)} + \mathbf{W_i^{(n,H)}H^{(t-1)}} \\ &\qquad + W_h^{(n)}(r^{(t)} \odot H^{(t-1)}) + b_n\Big). \end{aligned} \tag{19}$$

In a standard GRU, the gradient of the new state w.r.t the old state relies heavily on the active gates. If $z^{(t)}$ and $r^{(t)}$ saturate to 0, the gradient signal diminishes. In contrast, the term $\mathbf{W_i^{(n,H)}H^{(t-1)}}$ in our modified GRU acts as a direct gradient shortcut. Omitting the bounded derivative of the tanh function and considering $r^{(t)}$ as a constant factor, the partial derivative of $n^{(t)}$ w.r.t $H^{(t-1)}$ has a path independent from the reset gate $r^{(t)}$:

$$\frac{\partial n^{(t)}}{\partial H^{(t-1)}} \propto W_h^{(n)} \cdot \mathrm{diag}(r^{(t)}) + \mathbf{W_i^{(n,H)}} \tag{20}$$

Therefore, the gradient flow to $H^{(t-1)}$ always sustains even if the reset gate $r^{(t)} \to 0$, facilitating learning over multiple EGNN layers.

Interface-focused K-hop sampling

The interface-focused K-hop sampling algorithm outputs a subgraph centered around the interface region of a given input graph. This iterative algorithm starts from a set of seed nodes, which can be either CDR nodes or any nodes in inter-Ig-Ag edges. The output subgraph includes all sampled nodes and any edges established between them.

Algorithm 1 K-hop Subgraph Sampling

Require: $\mathcal{G}$, k, node threshold $N_{\max}$, optional seed set S
if $S = \emptyset$ **then**
  $S \leftarrow \{u, v \mid (u, v) \in \mathcal{E}_{\text{inter}}\}$ ▷ interface seeds
end if
$C \leftarrow S$ ▷ current selected nodes; $|S| < N_{\max}$ by assumption
for $i \leftarrow 1$ to k **do**
  $L \leftarrow \text{unique}(\text{BFS\_layer}(\mathcal{G}, S, i))$
  $L_{\text{new}} \leftarrow L \setminus C$
  if $L_{\text{new}} = \emptyset$ or $|C| + |L_{\text{new}}| > N_{\max}$ **then**
    break
  end if
  $C \leftarrow C \cup L_{\text{new}}$
end for
return subgraph($\mathcal{G}$, C)

Threshold Selection

Baseline methods output various ranges of continuous values with different meaning. We acknowledge that a robust classifier must have a unique threshold for all datasets. Therefore, we design **Algorithm 2** to select a classification threshold that maximize the F-beta score on the evaluation set. In practical virtual screening tasks, we can also opt for the Top-k thresholding approach to prioritize the most promising leads, ensuring high precision among the top-ranked candidates which are most likely to undergo further lead optimization.

Algorithm 2 Select threshold maximizing F-beta score based on the evaluation set

Require: $S = \{s_i\}_{i=1}^n$, $s_i \in \mathbb{R}$, $Y = \{y_i\}_{i=1}^n$, $y_i \in \{0, 1\}, \beta = 0.25$
$\mathcal{T} \leftarrow \text{unique}(S) \cup \{0, 1\}$ ▷ Candidate thresholds
$\text{score}^* \leftarrow 0, \ \tau^* \leftarrow 0$
for all $\tau \in \mathcal{T}$ **do**
  Predict $\hat{y}_i \leftarrow \mathbf{1}\{s_i \geq \tau\}$ for $i = 1, \ldots, n$
  $\text{score} \leftarrow (1 + \beta^2) \cdot \dfrac{P \times R}{\beta^2} \times P + R$
  if score > score* **then**
    $\text{score}^* \leftarrow \text{score}, \quad \tau^* \leftarrow \tau$
  end if
end for
return (τ^*, score^*)
